# A Comprehensive Narrative Review of the Impact of Pelvic Radiotherapy on Pelvic Bone Health: Pathophysiology, Early Diagnosis, and Prevention Strategies

**DOI:** 10.7759/cureus.66839

**Published:** 2024-08-14

**Authors:** Mohamed Elgendy, Alvin Billey, Asra Saleem, Bushra Zeeshan, Gayanthi Dissanayake, Meaza Zergaw, Marcellina Nwosu

**Affiliations:** 1 Trauma and Orthopedics, California Institute of Behavioral Neurosciences & Psychology, Fairfield, USA; 2 Pathology and Laboratory Medicine, California Institute of Behavioral Neurosciences & Psychology, Fairfield, USA; 3 Internal Medicine, California Institute of Behavioral Neurosciences & Psychology, Fairfield, USA; 4 Dermatology, California Institute of Behavioral Neurosciences & Psychology, Fairfield, USA; 5 Internal Medicine and Family Medicine, California Institute of Behavioral Neurosciences & Psychology, Fairfield, USA; 6 Medicine, California Institute of Behavioral Neurosciences & Psychology, Fairfield, USA

**Keywords:** intensity-modulated radiotherapy, bone density, pelvic malignancy, pelvic radiotherapy, insufficiency fractures

## Abstract

Radiotherapy is a commonly used modality in pelvic malignancies such as prostate, gastrointestinal, or gynecological, either as a primary treatment or an adjuvant post-surgery. Despite its positive impact on the prognosis of these patients, it was found in several studies that it contributes to insufficiency fractures in different sites of the pelvis, more commonly in the sacral ala. This is particularly true for elderly patients. There are several hypotheses on how radiotherapy affects bone health, as it destroys the bone matrix and causes obliterative vasculitis. Several imaging techniques, particularly magnetic resonance imaging (MRI), help detect the radiotherapy-induced fracture and distinguish it from metastases. Some modalities, such as intensity-modulated radiotherapy (IMRT) and brachytherapy, have decreased fracture risk by escaping the adjacent structures to the targeted organ. Pharmacological interventions such as amifostine and desferrioxamine are promising in terms of bone protection, which necessitates further studies to confirm their mechanism of action.

## Introduction and background

Radiotherapy (RT) is commonly used as a treatment modality, either as an adjuvant after surgery or as a primary treatment [1], for gynecological and gastrointestinal malignancies, as in cervical cancer, in which it is commonly used. It is also known for managing endometrial, anal, and rectal tumors [2]. Moreover, it has been relied on to prevent heterotrophic ossification, a total hip replacement complication [3]. Although it has improved the prognosis of these patients [1], it hurts bone density by negatively impacting bone minerality, which thins the bone and leads to sclerosis [4]. RT destroys osteoblasts by damaging their deoxyribonucleic acid (DNA), which disrupts bone synthesis by increasing cell apoptosis [3,5]. Consequently, it leads to pelvic insufficiency fractures, typically in the sacrum, which can occur even with minor trauma, thereby significantly increasing the patient's morbidity and mortality rate [6].

A mechanism by which it interferes with bone synthesis is that it decreases its blood supply [7]. A study by Tilman Bostel showed that RT can also be used as adjuvant therapy after surgery in sacral chondromas or even as a primary treatment for those who are not good candidates for operation or have refused it. However, RT can have toxic effects, resulting in neuropathies and long-term pain, in addition to the high risk of sacral insufficiency fractures [8], especially when using high-dose carbon ion-based RT in combination with surgical intervention [9].

Different imaging techniques are used to identify pelvic insufficiency fractures, such as the dual-energy X-ray absorptiometry (DEXA) scan, which measures bone density and assesses fracture risk [10]. Besides, magnetic resonance imaging (MRI) can help detect subtle fractures by detecting the fracture lines and also differentiating between the RT-induced insufficiency fracture and the malignancy-induced one, thus saving the patient an unnecessary bone biopsy, which can worsen the fracture or even cause bleeding [11], by being very sensitive to the reactive bone marrow changes accompanied by insufficiency fractures during the post-RT follow-up [12]. This article aims to provide helpful insight into the effect of pelvic RT on pelvic bone density, study pathophysiology, identify methods of early diagnosis and prevention, and discuss how we can avoid any delay.

## Review

Pathophysiology

Bone Mineral Deficiency and Vasculitis

Pelvic RT-related insufficiency fractures can dramatically affect elderly morbidity and mortality, particularly in women who have undergone pelvic radiation for a gynecological malignancy [13]. However, a separate study by Igdem et al. demonstrated that patients with prostate cancer who underwent pelvic RT also sustained pelvic insufficiency fractures [14]​. The femur, pelvic rami, and symphysis pubis are common fracture sites, and avascular necrosis of the femur's head is also a common risk [15,16]. Due to their proximity to the pelvic organs, the pelvic bones will also receive radiation. Several studies have investigated the pathophysiology of radiation-related IF. It has been known that radiation damages osteoblasts, osteocytes, and osteoclasts, diminishing the bone matrix and decreasing the functional component, making the bone more susceptible to fracture, especially in the weight-bearing areas [17]. As such, there is a decrease in collagen and alkaline phosphatase formation, which play crucial roles in bone mineralization [18]. It is suggested that this leads to osteopenia, weakening the bone's elastic resistance and decreasing its ability to bear the weight-bearing force [19]. All in all, there is a strong relationship between bone mineral density (BMD) and fracture susceptibility [20-22].

**Figure 1:**
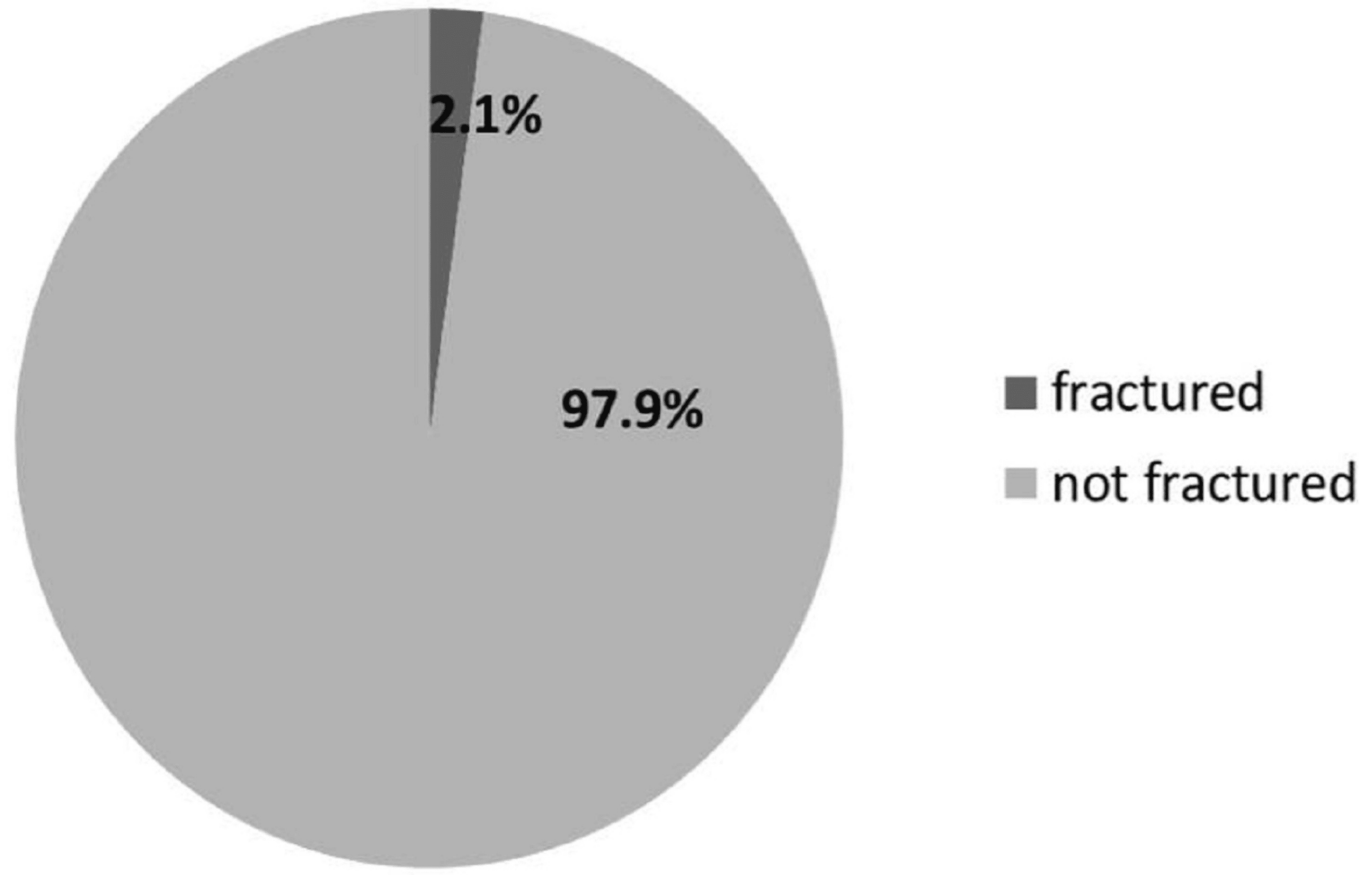
A pie chart that illustrates the 2.1% fracture risk in patients who received irradiation at a total dose of less than 50.4 GY. Data according to Oh et al.'s study (2008) [26]

A later effect of RT, first described by Ewing [23], is obstructive endarteritis and periarteritis, eventually resulting in endothelial cell swelling and vacuolation [18]. Given that these changes occur in the narrow Haversian channels, it is thought that this is how they cause connective tissue sclerosis. A much later sequel is the thickening of the hyaline cartilage of the blood vessel's tunica media due to subintimal fibrosis, ultimately narrowing the vessel lumen [24]. These are the typical features of the radiation-related changes in the other standard tissue systems [25]. Regarding bone density, a study found that the usual dose of RT, which is 45-50 gray (GY) after one to seven years, does not significantly affect BMD, contrary to what other studies concluded [10]. Moreover, a study by Oh et al. concluded a remarkable difference in the incidence of pelvis fracture caused by irradiation between patients treated with a total dose (TD) <50.4 GY and those treated with a TD ≥50.4 GY [26]. In patients who have undergone pelvic RT with a total dose of less than 50.4 GY, the incidence of pelvic fracture was 2.1% (Figure 1).

**Figure 2:**
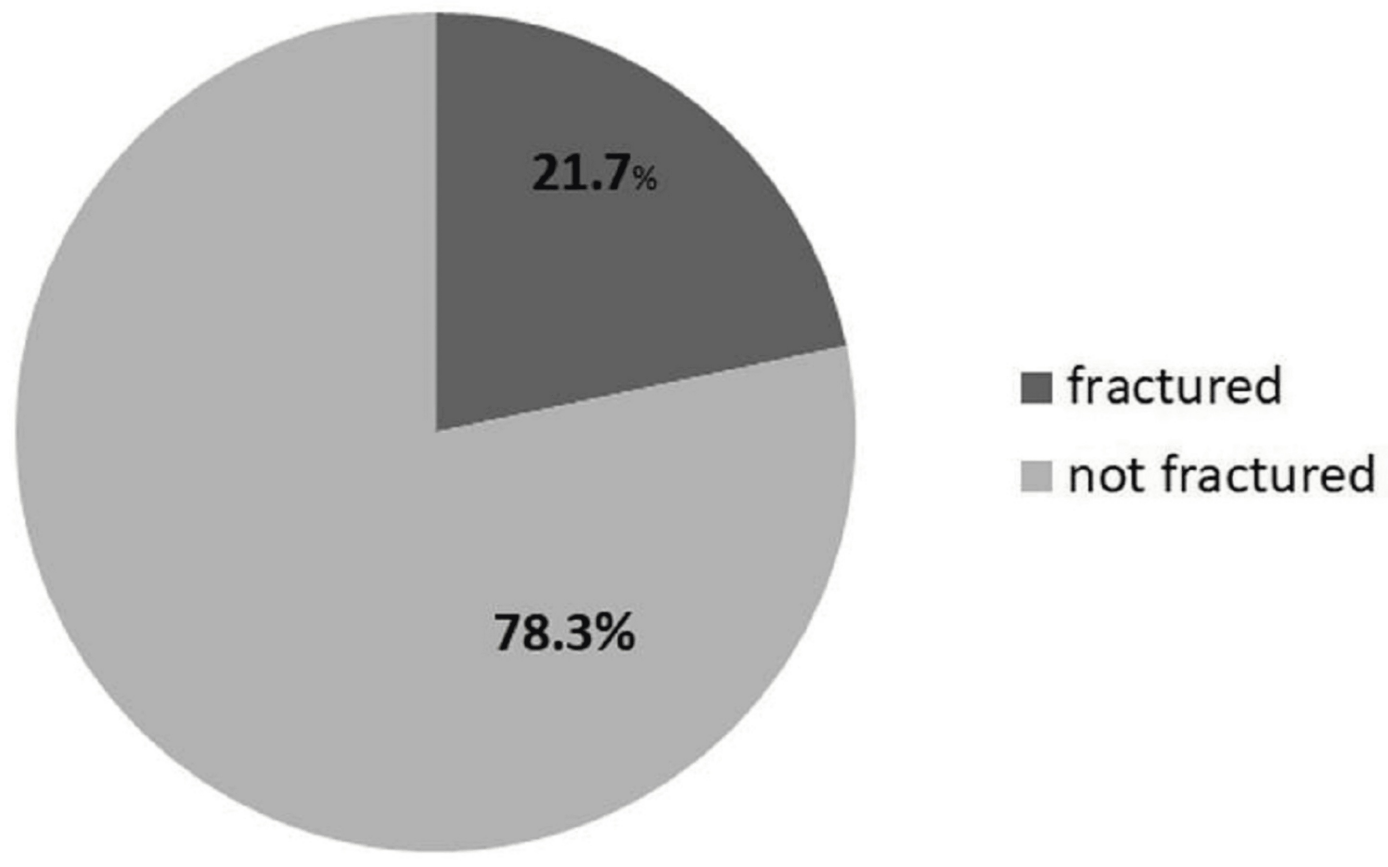
A pie chart illustrates that those patients who underwent irradiation at a total dose >/= 50.4 GY had a fracture risk of 21.7%. Data according to Oh et al.'s study (2008) [26]

In those who have undergone pelvic RT with a total dose of more than or equal to 50.4 GY, the risk of pelvic fracture was 21.7% (Figure 2).

**Figure 3:**
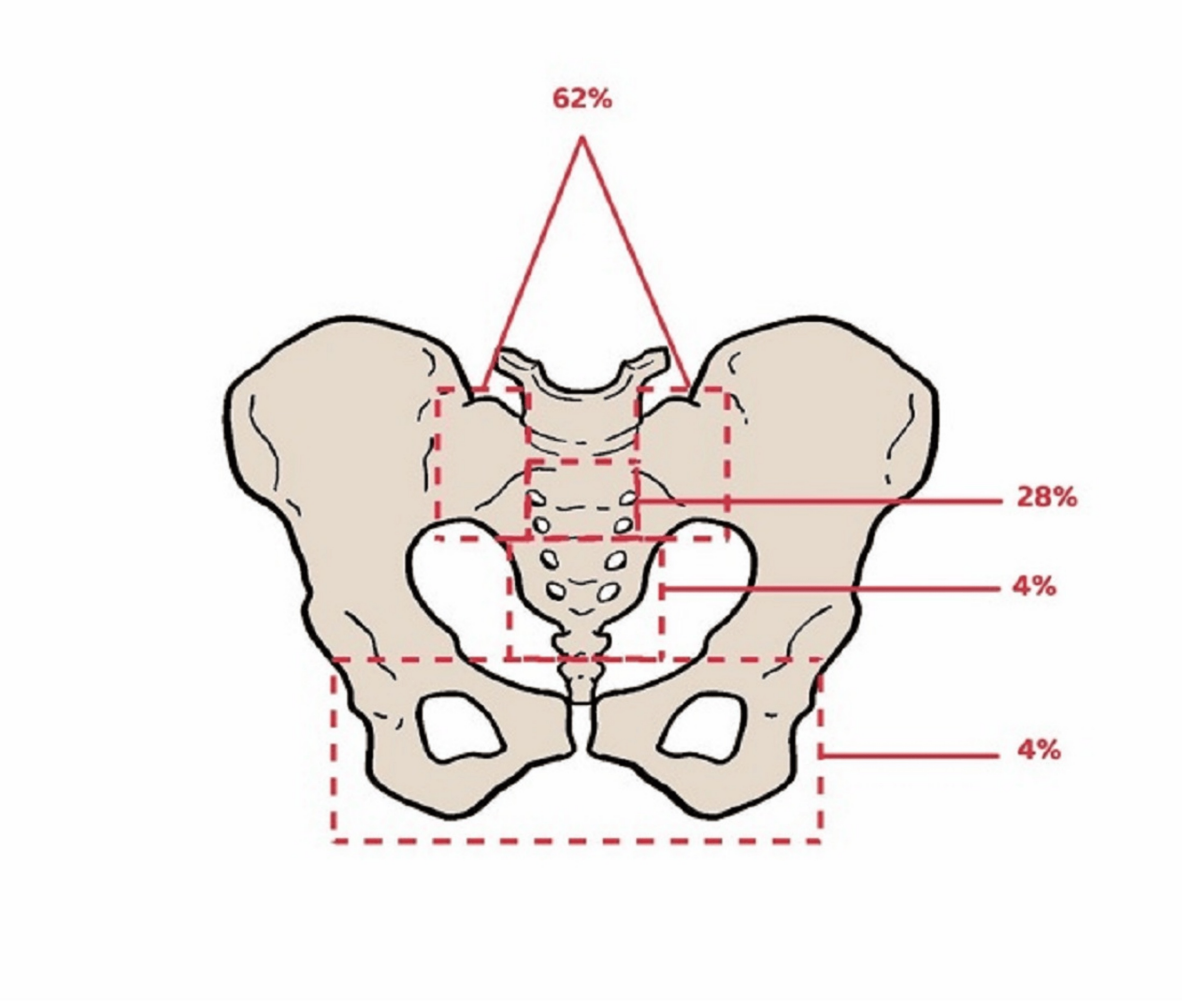
A diagram that illustrates the common sites of radiation-induced insufficiency fractures in the pelvis, along with the incidence of each fracture. The data are based on a study by Abe et al. (2013) [29]

**Figure 4:**
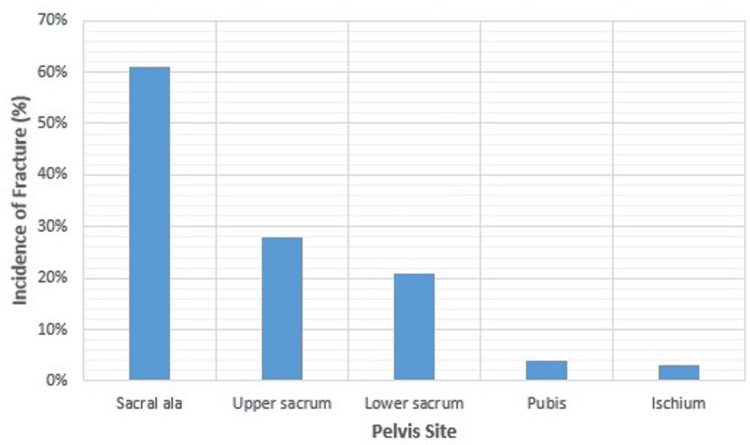
A graph that illustrates the common sites of radiation-induced insufficiency fractures in the pelvis, along with the incidence of each fracture. The data are based on a study by Abe et al. (2013) [29]

RT more commonly affects some pelvic parts, such as the sacroiliac joints, than others since each bone needs a different radiation dose to sustain irreparable osteoblasts, osteocytes, and osteoclast injuries [27]. For clarification, a bone with more loads can tolerate a lower radiation dose [28]. The sacrum, sacroiliac joints, and medial parts of the iliac bones are the best examples of the body's fundamental weight-bearing parts. A study by Abe et al. showed that the insufficiency fractures were being distributed among the pelvis as follows: 61% sacral ala and medial portion of the ilium, 28% upper sacrum, 21% lower sacrum, pubis 4%, and ischium 3% [29], as shown in Figures 3, 4.

Despite the association between BMD and bone fracture [20-22], another study showed that insufficiency fractures caused by RT are not related to the minerality of the cortical bone [30]. Thereby, it is thought that damage to the sacroiliac joint is more likely due to bone inflammation resulting from vascular insults than bone content deficiency [​16]​. The femur of the rats was investigated by Pitkanen et al. and Hopewell et al. for the effect of the irradiation, and it was shown that there was a change in the blood flow after being exposed to irradiation with single doses of 5-25 Gy. Additionally, the remarkable reduction in dry bone weight was seven months following exposure to radiation doses of 15, 20, and 25 Gy attributed to decreased blood flow that damaged the parenchyma or disrupted bone metabolism [31]. Consequently, a surgical complication of pelvic irradiation is post-total hip replacement implant failure [32]. A study by Vijayakumar et al. found that one patient in their cohort suffered aseptic acetabular component loosening after receiving one month of post-surgical RT. Furthermore, two more patients suffered hip dislocation, with one having undergone one month of irradiation and the other receiving two months of RT before the THA [33].

Diagnosis

Bone Scan, CT, and MRI

Two-thirds of patients with pelvic insufficiency fractures show ambiguous, nonspecific symptoms [34], such as lower spinal and buttock aches [35]. While most of the patients with pelvic insufficiency fractures are symptomatic, some are asymptomatic. In either case, it is crucial to investigate the patients undergoing pelvic RT to detect the fracture and its cause as quickly as possible to improve their prognosis [29]. A couple of studies investigated the different tools used to diagnose the fracture, one of which was the Technetium-99m (Tc 99m) bone scan. This scan is very sensitive as it shows increased activity in the pelvis symmetrically, giving the typical H shape that indicates fractures in both sacroiliac joints bilaterally and the body of the sacrum [36,37]. However, it also shows an asymmetrical increase in pelvis activity across multiple foci [​29]​. However, a drawback of the bone scan is that the H sign can sometimes be absent [38].

**Figure 5:**
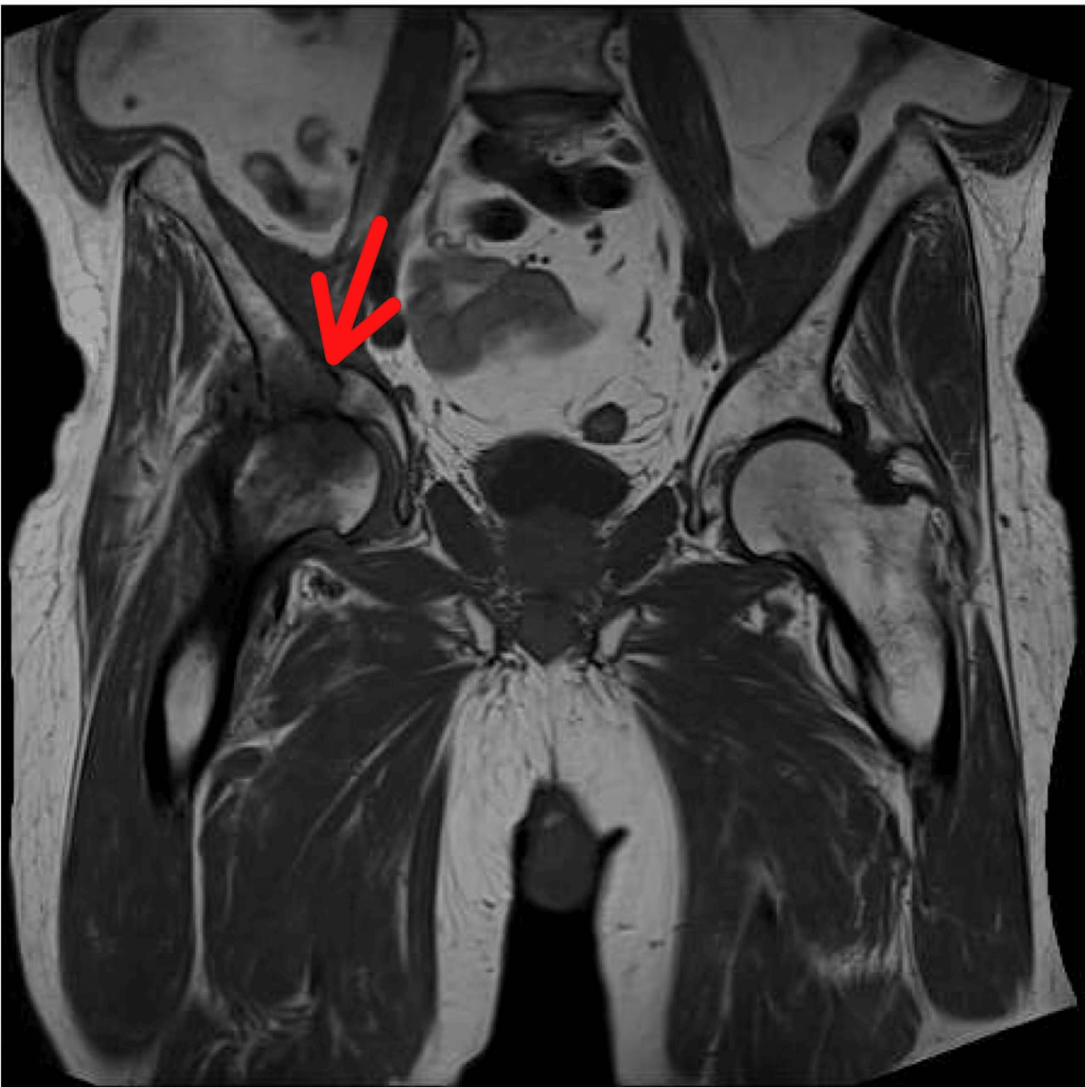
MRI pelvis showing a fracture of the right acetabulum (red arrow) after radiotherapy for prostate cancer. The examination was performed on October 27, 2023, in the radiology department at Bolton NHS Trust, UK.

Moreover, CT has shown a remarkable ability to delineate the fracture lines and differentiate the radiation-induced fractures from the ones caused by metastases, with a sensitivity of 60%-75% [39],​ even though MRI is proven to be the most sensitive, with a sensitivity of up to 100% since it images the soft tissues with high quality and captures the bone marrow edema caused by the fractures [11,12,19,34,39,40]. The following MRI scan shows a fracture of the right acetabulum after the patient has undergone RT for prostate cancer, according to the data collected from the radiology department at Bolton NHS Foundation Trust, UK (Figure 5).

**Figure 6:**
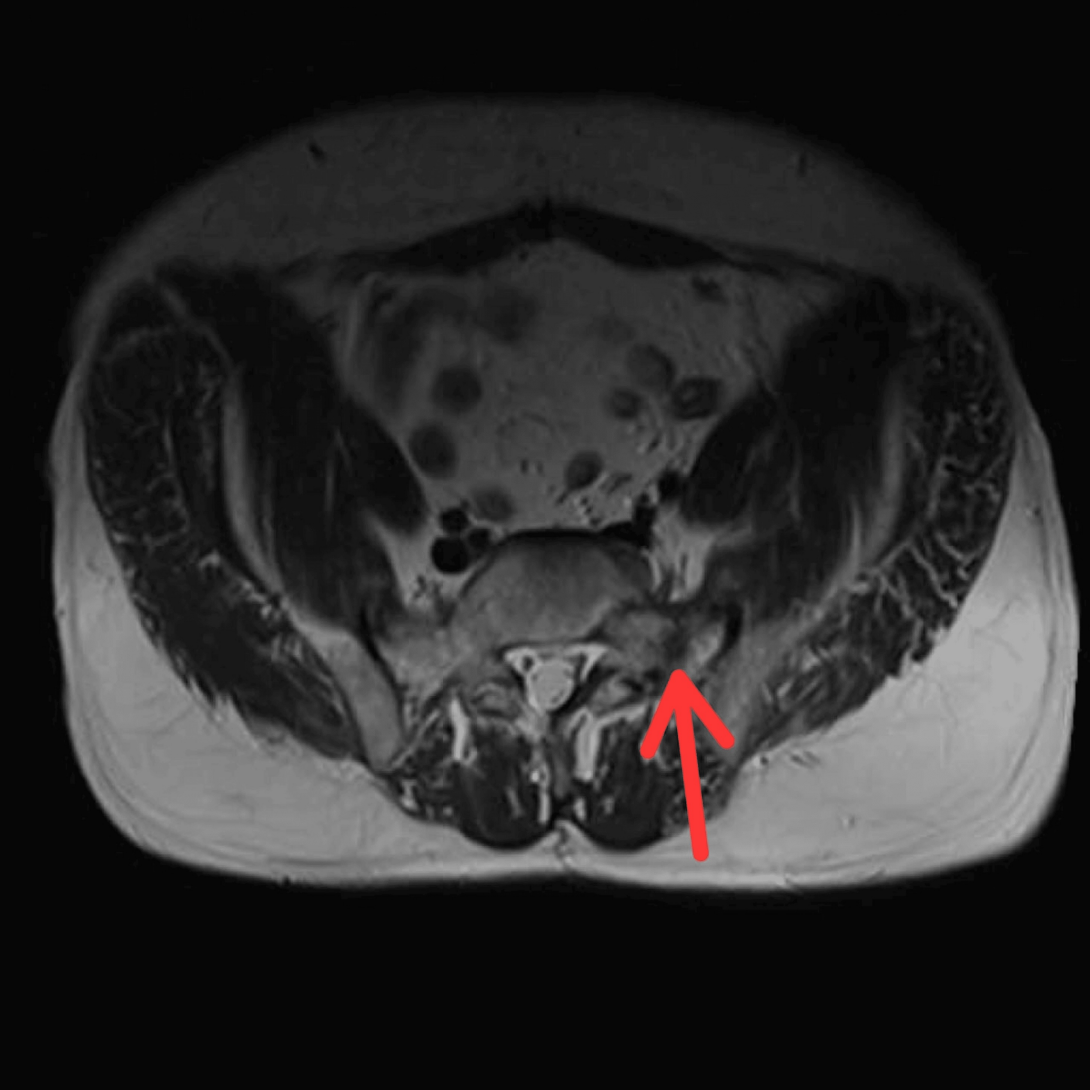
MRI showing a non-displaced fracture of the left sacral ala (red arrow) after radiotherapy to the rectum for rectal cancer. The examination was performed on October 3, 2022, in the radiology department at Bolton NHS Trust, UK.

Additionally, the following MRI scan shows a fracture of the left sacral ala after the patient has undergone pelvic RT for rectal cancer, according to the data collected from the radiology department at Bolton NHS Foundation Trust, UK (Figure 6).

Consequently, it can differentiate radiation-induced from metastatic fractures by ruling out the localized soft tissue mass surrounding the fracture sites [41,42]​. Furthermore, researchers found that some MRI sequences performed better than others. The coronal fat saturation FS-T2W is more accurate in detecting bone marrow edema and fracture lines than either T1 W1 or enhanced T1 W1, and thus, it should be the MRI sequence of choice when a pelvic fracture is suspected. Still, it can be seen on conventional radiographs [34,43].

Management and future recommendations

Pharmacological Interventions and Modified RT

Qurrat U van den Blink et al. conducted a study investigating pharmacological interventions that can reduce the risk of pelvic insufficiency fractures. The study found neither zoledronate nor other pharmacological interventions demonstrated any protective effect on the bones from irradiation. However, zoledronate may boost BMD and aid in androgen deprivation-specific bone loss, not RT-specific bone loss. However, some medications, such as amifostine and desferrioxamine, have shown some promise. Amifostine was found to be a radioprotective medicine by maintaining vascularity and enhancing bone repair following radiation treatment. Desferrioxamine has produced much higher callus size, strength, and mineralization than irradiated fractures [44]. Research has demonstrated that intensity-modulated radiotherapy (IMRT) may help reduce the incidence of pelvic insufficiency fractures by avoiding the bones and reducing the risk of blood toxicity, except for those over 50 and post-menopausal women [2]. On the other hand, it is worth mentioning that Shih et al. concluded that there is no apparent difference between patients treated with three-dimensional (3D) conformal RT and those treated with IMRT [45]. Vitzthum et al. reported that brachytherapy also showed a decreased risk of pelvic fracture, which is optimum in the first two years. However, it is not yet understood why both IMRT and brachytherapy only reduce the risk of fractures in women and not in men [​6].​

Pain Management and Pre-exposure Investigations

Nonsteroidal anti-inflammatory drugs, paracetamol or analgesics such as weak opioids, and rest are management options for radiation-induced insufficiency fractures [40,46,47]. However, hospital admission and patient management might be needed for severe pain that requires specialized treatment [​35,46]. Furthermore, other investigations can be helpful, such as bone density assessment by densitometry before exposure to RT, which decreases fracture susceptibility. The bone profile, which includes calcium, phosphorus, vitamin D, alkaline phosphate, and albumin, can also assist in systemically measuring bone minerals [2]​.

## Conclusions

This article examines the effect of pelvic radiation on the pelvic bones and explores potential preventative strategies. RT is commonly used either as a primary treatment or an adjunct. However, pelvic insufficiency fractures are not uncommon complications that occur after pelvic RT. However, they can be misinterpreted as metastasis-induced fractures or because they are too subtle to be detected by conventional radiography. Therefore, CT scans and radionuclide bone scans can be used. The latter may display an H-shaped pattern. However, this pattern may be absent in certain instances. That is when the role of the MRI comes in, which is shown to be the most sensitive imaging modality by showing focal edema around the fracture site.

Regarding protective strategies, IMRT and brachytherapy have demonstrated the ability to spare the bones and only target the afflicted organ. It is imperative that we take this fact into account and conduct further studies. However, additional studies and investigations are needed to understand why this only applies to women, not men. Amifostine and desferrioxamine are the pharmacological interventions that have shown promise in bone protection for RT patients, and they need further investigations to confirm their role. Furthermore, some blood tests, such as a pre-radiation bone profile and densitometry analysis of the bone density, are recommended. Thus, the health practitioner will be able to estimate the likelihood of the patient developing a fracture as a sequel, balance the advantages against the disadvantages, and, finally, determine whether irradiation is indeed necessary.
